# Intensive Value Utilization of Food‐Derived Marine Immunoactive Peptides: Optimizing the Process Yield and Improving the Delivery Efficiency According to the Immune Activity Mechanism and Assisted Enzymatic Hydrolysis

**DOI:** 10.1002/fsn3.70578

**Published:** 2025-08-05

**Authors:** Zhicheng Yin, Yingying Tian, Shuteng Huang, Hong Wang, Lili Zhao, Ruyue Zhang, Desheng Cai, Shuping Wang, Shaojing Zhong, Jiayu Zhang

**Affiliations:** ^1^ School of Traditional Chinese Medicine Binzhou Medical University Yantai China; ^2^ College of Pharmaceutical Science Shandong University of Traditional Chinese Medicine Jinan China; ^3^ College of Life Sciences Shandong Agricultural University Taian China; ^4^ Yantai New Era Health Industry Daily Chemical Co., Ltd Yantai Shandong China

**Keywords:** food‐borne immunoreactive peptides, immune regulation, preparation process, structure–activity relationship

## Abstract

Food‐borne marine active peptides have been increasingly used as functional products for postoperative rehabilitation. However, there are still many problems, such as low protein yield and absorption rate. Therefore, the assisted enzymatic hydrolysis process and activity of marine immunoreactive peptides have been the focus of widespread attention. In particular, they are used as natural immunomodulators to improve intestinal microbiota and immune damage. Most peptides are barely digested by the gastrointestinal tract, especially in the immune microenvironment‐damaged intestine. How to make the damaged intestine fully absorb active substances has become an urgent problem to be solved in food distribution. Therefore, the optimization process method is summarized to improve the quality and efficiency, and according to the immune activity mechanism combined with peptide self‐assembly food application, which provides an alternative for the design of functional products. Sustained release of peptide‐derived hydrogels increases their retention time in the body and promotes complete absorption in the gastrointestinal tract. By targeting release and enhancing biologically active functions, peptides avoid enzymatic hydrolysis in the gastrointestinal tract, thereby improving the stability of food‐derived immunoactive peptide delivery. Moreover, with the increasing correlation between food‐borne products and intestinal flora, the active sites of food‐borne immunoactive peptides and metal ion chelating peptides are more conducive to the micro‐regulation of intestinal flora. This review provides an opportunity for food and drug applications and development related to immunotherapy.

## Graphical abstract

To investigate the impact of assisted enzymatic hydrolysis on enhancing protein yield and biological activity. The analysis of the potential mechanism of intestinal immune regulation mediated by intestinal microorganisms is conducted. This article provides a review of the potential uses of functional, immunoactive peptides in food delivery and offers valuable insights for forthcoming product development and utilization.

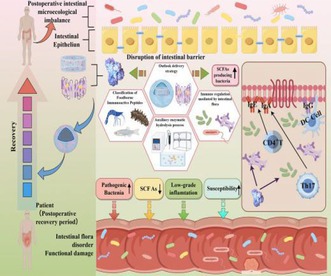

## 1 Introduction

**FIGURE 1:**
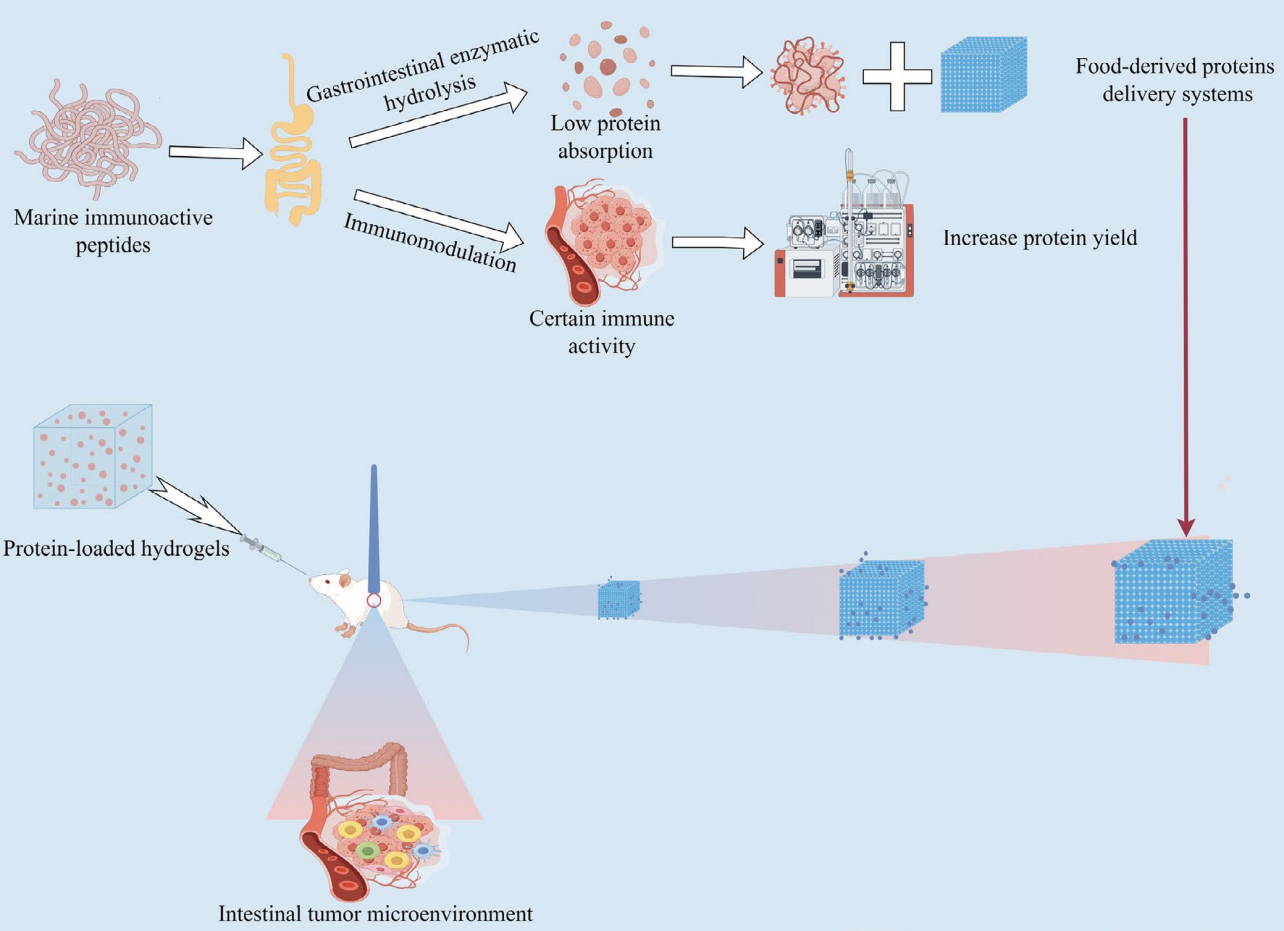
Research status of food‐borne marine immunoactive peptides (by Figdraw).

As interventional immunotherapies, immunomodulators can enhance or inhibit host defense by effectively alleviating, inhibiting, and up‐regulating the biological or synthetic origin of any component of innate or adaptive immunity. Metabolites are referred to as immunomodulators (Wu et al. 2020). At present, immunotherapy activates autoimmune response by regulating immune checkpoints, but there is still a risk of recurrence and organ lesions (Zhao et al. 2012). Since most immunomodulators are associated with some organ damage, the incidence of which is second only to chronic diseases (Ponticelli and Glassock 2019). For tumor treatment, it is not only necessary to control tumor cells but also to inhibit the production of complications and the proliferation of cancer cells by regulating the intestinal microenvironment, thereby reducing the immune damage caused by immunotherapy. Immunoactive protein products in marine foods have attracted much attention due to their small side effects and high efficacy. As shown in Figure 1, metal ion chelation or oral administration in the form of hydrogels can increase the abundance of beneficial bacteria in the intestine and further improve the intestinal mucosal barrier (Ahmad et al. 2019). The coating made from clickable mussel‐derived peptides can induce Foxp3^+^regulatory T (iTreg) cells to have a synergistic effect and promote osteogenesis (Zhao et al. 2021). It can be seen that marine immunoactive peptides can not only improve the tumor microenvironment but also improve the immune microenvironment of the body and enhance the postoperative recovery effect of patients.

However, there are deficiencies in the utilization and absorption of natural products. Therefore, how to optimize the preparation process and improve the delivery efficiency based on the immune activity mechanism has become an urgent problem to be solved. As shown in Figure 1, it is reported that the absorption rate of marine immunoactive peptides is relatively low (Ma et al. 2018). Marine bioactive peptides effectively activate the immune system to regulate the intestinal immune microenvironment through drug delivery systems such as hydrogels, stimulate the initial immune response by improving the intestinal flora, thereby modulating dendritic cells (DC), macrophages (Macrophages) and intestinal mucosal‐specific immunity, and ameliorating immune‐damaged diseases (Ahn et al. 2015; Bersch et al. 2021). In addition, the enrichment of marine biological proteins can increase the utilization rate of immunoreactive proteins, thereby enhancing the function of immunoreactive peptides to improve intestinal flora disorders and immunodeficiency.

## 2 Preparation Process of Food‐Borne Marine Immune Response Peptides

At present, bioengineered immunoreactive peptides can directly or indirectly bind to and kill cancer cells, but it is worth noting that G0 latent cancer cells are still undetectable in the late stage of tumor treatment (Li et al. 2023). Only by enhancing the activity of T cells in the late stage of immunotherapy can the immune damage be effectively improved. Bioactive peptides extracted from food proteins are highly active and easily absorbed (Anjum et al. 2017; Chalamaiah et al. 2018). They can completely pass through the intestinal barrier and exhibit biological functions at the tissue level. It can be used as an immunomodulator for interventional therapy. The preparation process is mainly related to the influence of biological factors (protease type) and physical factors (ultrasound, pulsed electric field) on protein yield. For industrial production, biological and physical factors combined with enzymatic hydrolysis to improve quality and efficiency may be more conducive to improving protein yield. Further exploration and optimization of the preparation process of bioactive peptides will provide lower production costs and higher and better protein yield for large‐scale production.

### 2.1 Vertebrates

#### 2.1.1 Mackerel‐Tuna

**FIGURE 2:**
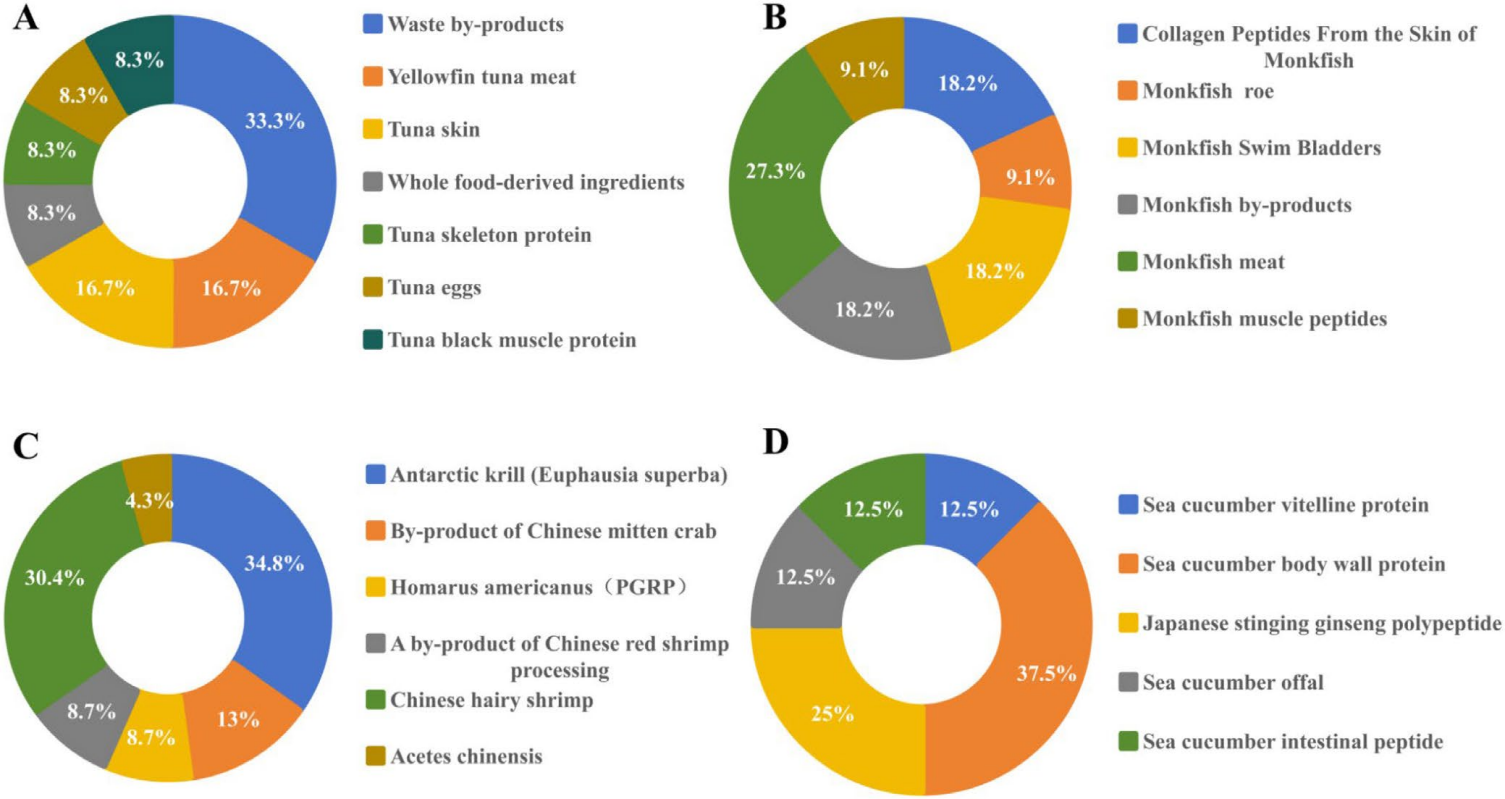
Source site of food‐borne marine immune active peptide (A: Tuna; B: *Lophius* little; C: Shrimp; D: Sea cucumber).

As shown in Table 1, Tuna meat oligopeptide (TMOP) was found to exert antihyperuricemic blood syndrome and kidney inflammation by mediating intestinal microbiota (Han et al. 2020), indirectly affecting the response mechanism of the immune system. The enzyme peptide from tuna effectively regulates inflammation and intestinal flora in DSS‐induced UC model mice (Kim et al. 2021). As shown in Figure 2A, tuna active peptides have been extracted from a variety of sources, ranging from skin to fish eggs (Harnedy et al. 2018). In the past 10 years, the research on tuna processing waste and surimi has been increasing, indicating that the current research on marine organisms has begun to develop towards the intensification and enrichment of marine biological products by converting wastes into productive materials (Cai et al. 2022). Meanwhile, studies have found that the antioxidant peptides ICRD and LCGEC possess immunomodulatory activity, confirming the immunomodulatory role of active antioxidants (Han et al. 2020). Thus, it is evident that proteins from tuna fish protein to fish egg protein have significant potential as immunomodulatory functional foods.

**TABLE 1:** Summary of marine animal‐derived immunoreactive peptides and their hydrolysates related to intestinal flora or cytokines in the treatment of diseases.

Source	Source of enzymatic hydrolysis	Peptide/Hydrolysate	Polypeptide sequence	Efficacy results	Protease type	Molecular weight	Cell cytokine/Signaling pathway	Target/Disease	References
Tuna	Whole derived food‐derived ingredients	Tuna meat oligopeptide (TMOP)	PPCQLINQTVS	in vivo experiments	Trypsin and alkaline protease (3%)	< 1 kDa	Inhibition of NLRP3 inflammasome and TLR4/MyD88/NF‐κB signaling pathway activation, inhibition of NF‐κB p65 phosphorylation	Hyperuricemia and associated renal inflammation	Han et al. 2020
	Tuna dark meat	Tuna meat hydrolysate (TPH)			Trypsin	4.7–6.5 kDa		Hyperuricemia	Zhang et al. 2019
	Tuna Muscle Tissue	Tuna black muscle protein (TDMP)	KKLGELLK and KLGELLK	in vivo experiments (0.5 mg·mL‐1)	Trypsin	3 kDa	Activation of peroxisome proliferator‐activated receptor‐α expression in the liver was enhanced carnitine palmitoyltransferase‐2 activity	Obesity and other related diseases	Maeda et al. 2017
	Tuna processing waste	Tuna Bioactive Peptide (TBP)		In vivo experiments	Pepsin	< 1 kDa	The expression of inflammatory factors (LPS, IL‐6 and TNF‐α) was reduced	Colitis	Xiang, Zheng, et al. 2021; Xiang, Zhou, et al. 2021
	Tuna bone	Tuna skeleton protein (APTBP)	GRKKRRQRRRPPQVKAGFAWTANQQLS	In vitro and in vivo experiments	Trypsin	0.25–1 kDa	The transcription and release of inflammatory cytokines were reduced, and the activation of NF‐κB and the expression of inflammatory cytokines were significantly reduced	Inflammation of necrotizing enterocolitis	Zhang et al. 2019
	Tuna eggs	Novel peptides Ile‐Cys‐Arg‐Asp (ICRD) and Leu‐Cys‐Gly‐Glu‐Cys (LCGEC) were synthesized	Ile‐Cys‐Arg‐Asp (ICRD) and Leu‐Cys‐Gly‐Glu‐Cys (LCGEC)	In vitro and in vivo experiments	Trypsin and alkaline protease	≤ 2 kDa	Keap1/Nrf2‐ARE pathway transcription was changed, Keap1/Nrf2‐ARE pathway was regulated, and the release of pro‐inflammatory cytokines was inhibited	Inflammation	Han et al. 2020
Lophius	Anglerfish meat	2,2‐Diphenyl‐1‐nitro phenylhydrazine [lophius little peptides (LPs)]		In vivo experiments	Neutral protease	< 1 kDa	Improve antioxidant activity and reduce the level of inflammatory cytokines. TLR4/NF‐κB signaling pathway was inhibited	Renal injury and inflammation caused by lipotoxicity	Mackin et al. 1991
	Skipjack by‐products	Preparation and purification of skipjack tuna enzyme peptide (SEP) from skipjack tuna by‐products		In vitro experiments			The levels of inflammation‐related IL‐6, IL‐10, and TNF‐α were decreased	Inflammation, ulcerative colitis	Wang et al. 2021
Shrimp	Antarctic krill	Antarctic krill (AKP) peptide		In vitro and in vivo experiments	Trypsin		The protein expression on the Nrf2/HO‐1 single pathway was activated, and the Nrf2/HO‐1 pathway was activated	Acute liver injury	Wang et al. 2021
	Shrimp	Shrimp peptide hydrolysate (SPH)		In vitro and in vivo experiments	Chymotrypsin	< 10 kDa	Increased immune organ index, serum cytokine levels (IFN‐γ, IL1β, TNF‐α, IL‐6), and immunoglobulin levels (IgA, IgM)	Cancer immune damage	Khan et al. 2022
Sea cucumber	Sea cucumber intestinal hydrolysates	Sea cucumber intestinal peptide (SCIP)		In vitro and in vivo experiments	Alkaline protease	< 1 kDa	Inhibition of the PI3K/AKT signal transduction pathway promotes the expression of related apoptosis‐related proteins	Antioxidant, antibacterial and antitumor effects, cancer	Wei et al. 2021
	Sea cucumber	Sea cucumber peptide (SCP)		In vitro and in vivo experiments			Upregulate the immune response of T lymphocyte subsets and increase the proportion of CD4+/CD8 + T lymphocytes	Immune response to antiallergic therapy	Chen et al. 2025
	Sea cucumber enzymatic hydrolysates	Sea cucumber hydrolysates (EH‐JAP and EH‐LEU)	VPSLGVKMDAVPGR	In vitro and in vivo experiments	Trypsin and alkaline protease		Up‐regulate the transcription of anti‐inflammatory cytokines and inhibit the activation of TLR4/MyD88/NF‐κB signaling pathway	Hyperuricemia and Renal Inflammation	Wan et al. 2020
	Protein hydrolysates	Sea cucumber protein hydrolysate (antioxidant peptide)		In vitro and in vivo experiments	Trypsin and papain		Scavenging‐free radicals	Anti‐aging, anti‐oxidation	Gao et al. 2020
	Frondanol A5	Sea cucumber extract		In vitro and in vivo experiments			The increase of cytokines, inflammatory cytokines, increased GILT expression, and macrophage phagocytosis	Intestinal tumors, chronic inflammation	Ismail et al. 2025
Oyster	Hydrolysis of oyster tissue lysates	Oyster polypeptide (OP)		in vitro and in vivo experiments	Neutral protease		The expression of PEPCK and AMPK was up‐regulated	Anti‐fatigue effect	Wan et al. 2020
	Oyster protein	Oyster hydrolysate	VLDSGDGVTH	cell、in vitro and in vivo experiments	Trypsin	> 3 kDa	The growth of tumor cells was inhibited and the activity of NK cells was significantly increased	Anti‐tumor and immunostimulatory effects	Wang et al. 2010
	Oyster protein	Oyster peptide		In vitro and in vivo experiments			It stimulated the secretion of cytokines and promoted the relative mRNA levels of Th1/Th2 cytokines (IL‐2, IFN‐γ, IL‐4, and IL‐10)	Immune diseases, cancer	Xiang, Zheng, et al. 2021; Xiang, Zhou, et al. 2021

Based on ensuring “efficiency”, the optimization of “quantity” should also be realized. Traditional enzymatic extraction technology may lead to a low yield of tuna collagen (Benjakul et al. 2010). Microwave‐assisted hydrolysis for preparing antioxidant peptides (Lin et al. 2019) reduces the reliance on animal proteases and helps to lower costs. As shown in Table 2, ultrasonic technology can improve protein extract ability by accelerating phase transition or reducing particle size. With the increase of ultrasonic time, it was found that the content of proline and hydroxyproline in collagen increased significantly compared with traditional enzymatic hydrolysis (Pezeshk et al. 2022), resulting in a high thermal denaturation temperature and good stability of collagen. Furthermore, the formation of pyrrole rings, hydrogen bonds, and the presence of glycine in tuna collagen contribute to strengthening its helical structure (Wu et al. 2020), minimizing inefficiencies in the preparation process. However, it is important to control the ultrasonic treatment time to prevent protein modification and activity reduction. Collagen can also be extracted from marine by‐products such as tuna skin by relatively mild methods such as isoelectric precipitation (Lin et al. 2019). Currently, this approach has been applied in the pilot‐scale processing of catfish fillet products (Mireles DeWitt et al. 2007). It is clear that the nonthermal‐assisted technology not only retains the protein activity but also improves enzymatic hydrolysis efficiency and protein yield, offering promising prospects for food industrialization.

**TABLE 2:** Research progress of using auxiliary technology to improve the production'quantity” and'efficiency” in the process of proteolysis.

Food‐borne marine animal products	Source site	Protease	Operation conditions	Conclusion	References
Tuna	Fish skin (collagen)	Pepsin	Isoelectric precipitation method:pH 2.0–3.0; the temperature is 4°C; time 24–48 h.	The yield of the PSC‐IP product was about 17.17% (dry weight), which was higher than that of PSC‐SO (14.14% dry weight)	Lin et al. 2019
	Fish skin (collagen)	Pepsin	Ultrasonic treatment:Frequency 20 kHz, power 200 W, time 30 min; pH 3.0	Extraction efficiency increased 2.7×; preserved triple‐helix structure; enhanced solubility	Pezeshk et al. 2022
	Fish meat	Neutral enzyme	Microwave pretreatment; Power 600 W, time 5 min; pH 7.0; enzyme concentration 2%	Neutral enzymatic hydrolysates with the highest degree of hydrolysis (29.54% ± 1.19%) and antioxidant activity were isolated by ultrafiltration and a series of chromatography	Wang et al. 2021
	Twisted scraps	Alkaline protease	Myeloperoxidase; pH 9.0; the temperature is 50°C; time 4 h; enzyme substrate ratio 1:20	The potential peptides inhibiting MPO activity were screened according to the docking energy	Cai et al. 2022
	Muscle	Alkaline protease	Homogenization conditions: speed 10,000 rpm; degreasing: isopropyl alcohol soaking, time 2 h	The results indicate that the defatted DMT contains high levels of Se in a nutritionally available form	Zhao et al. 2024
	Blood	Neutral enzyme	Pretreatment: 95°C heating for 15 min; pH 7.0; enzyme concentration was 1.5%.	The addition of mango jelly showed strong antioxidant and ACE inhibitory activity with the increase of hydrolysate	Zheng et al. 2022
Shrimp	Shrimp by‐products (shrimp head)		Ultrasound‐assisted: frequency 40 kHz, time 20 min; temperature 45°C	Ultrasonic‐assisted extraction increased the content of taste‐active compounds in WSHs	Duppeti et al. 2023
	Shrimp by‐products (head, chest, shell and tail)	Endogenous protease	Low temperature protection conditions: temperature 4°C; time 12 h	Antifreeze peptide activity, calcium ion ATPase activity, with low‐temperature protection potential	Duppeti et al. 2023
	Shrimp by‐products (shrimp head)	Endogenous protease	UV treatment: wavelength 254 nm, time 15 min; gradient temperature: 30°C → 50°C, total time 5 h	After 5 h of autolysis, the protein recovery of the UV‐heat‐treated samples was up to 92.1%. Combining UV irradiation with gradient temperature improves the protein recovery from shrimp head waste	Zhu et al. 2021
	Shrimp by‐products (cephalothorax, shells and polypods)	Endogenous protease	Autolysis hydrolysis: temperature gradient (30°C → 60°C), total time 6 h; pH 7.5	The hydrolysis produced at fixed and gradually increasing temperatures (GIT) was rich in small peptides that help to increase the value of Pacific white shrimp by‐products in the production of protein hydrolysates in autolysis hydrolysis technology	Cao et al. 2014
	Shrimp boiling water (SBW)	Endogenous protease	Pretreatment: Cara flocculation (45.4 g/L); the temperature is 25°C; time 30 min	Carrageenan (45.4 g/L) flocculation showed the most effective protein sedimentation, providing 78.5% protein recovery and 75% protein weight dry‐weight biomass	Wang et al. 2021
Sea cucumber	Sea cucumber all	Bromelain + papain or alkaline protease	Combined hydrolysis of bromelain and papain: pH 7.0; the temperature is 50°C; enzyme ratio 1:1; time 6 h	The hydrolysate of bromelain + papain contained higher concentrations of essential amino acids and the highest protein yield	Vu et al. 2023
	Sea cucumber viscera	Alkaline protease, trypsin, and flavor zyme flavorsome	Hydrolysis of different protease combinations:pH 8.5; the temperature is 55°C; enzyme concentration 2%; time 4 h	Optimized antioxidant activity (DH 19.08%) and functional properties Regarding functional properties, hydrolysates derived from neutral and flavor enzymes showed better foaming properties. Amino acid composition showed that papain, neutral enzyme, alkaline protease, and bromelain endowed the hydrolysate with high nutritional quality	Zhao et al. 2012
Oyster	Dried oyster soft tissue	Pepsin, trypsin, and Maxipro enzymatic hydrolysis	Hydrolysis of different protease combinations: Two‐step enzymatic hydrolysis: Pepsin:pH 2.0, 37°C, 2 h; 2. Trypsin:pH 8.0, 37°C, 4 h	In stimulated RAW 264.7 cells, these four proteolytic components showed excellent anti‐inflammatory activity.	Shen et al. 2023
	Meat		High hydrostatic pressure: Pressure: 200–500 MPa Time: 5–15 min ‌Temperature‌: 25°C or 4°C	There was no significant difference in the percentage of salt‐extractable protein (SEP) and total lipid values. High hydrostatic pressure extends the physical, microbial, and chemical quality of oysters	Li, Du, et al. 2022; Li, Li, et al. 2022
	Meat		Glycosylation Conditions‌: Glucose addition		Li et al. 2023
Other invertebrate marine animal shellfish	Oysters	Papain	pulsed electric field	Compared with other extraction methods, PEF accelerated the extraction speed and significantly improved the extraction rate of mussel protein. The protein yield reached 77.08%	Zhao et al. 2021
	Freshwater mussels	Flavor enzyme and trypsin	pulsed electric field	Improve solubility (91.54%) and emulsifying properties, The improvement of solubility (91.54%) and the decrease of viscosity by pulsed electric‐assisted field‐assisted enzymatic hydrolysis had a good effect on the improvement of the solubility of spices.	Zhao et al. 2021
Other vertebrate marine animals fish	Abalone viscera		Membrane ultrafiltration treatment: Membrane Cutoff: > 10 kDa	The retention rate of protein/peptide was > 10 kDa	Ghelichi et al. 2018
	Cod blood	Enzyme mixture	Ultrafiltration membrane electrodialysis	Enzymatic hydrolysis enhances angiotensin converting enzyme (ACE) inhibitory activity. .	Yamanushi et al. 2022
	Herring meat hydrolysate	Alkaline protease	centrifugation		

#### 2.1.2 Anglerfish

Ankang fish protein can serve as a source of high‐quality food‐derived bioactive peptides. As depicted in Figure 2B, Lophius little peptides are mainly derived from Lophius little meat protein, Lophius little by‐products, especially Lophius little skin, which is often discarded in food. Its collagen peptide exhibits a range of activities, including antitumor, antiulcer, antiviral, and other immunomodulatory activities (S.‐L. Zheng et al. 2022). As shown in Table 1, the Lophius little peptides have a small molecular weight, high safety, good stability, and are easily absorbed by the human body, making them highly favored by consumers in recent years. In terms of efficacy, Lophius little peptides have demonstrated a protective effect on renal injury and the potential to regulate intestinal microflora. After evaluating the results of oxidative stress, inflammatory factors, and intestinal flora experiments, Ankang fish protein significantly improves antioxidant activity and reduces inflammatory cytokine levels (David et al. 2014). Nowadays, diet is recognized as a key lifestyle factor that affects the intestinal flora, and Lophius little peptides can be used as an adjunctive therapy during the treatment of immune diseases. In terms of the preparation process, as shown in Table 1, most Lophius little peptides are hydrolyzed by neutral protease, whereas the most suitable choice for fish protein hydrolysis is an alkaline protease (Liu et al. 2013). Unfortunately, due to the pollution caused by enzymatic hydrolysis and the change in configuration, this method is not ideal for the commercial production of targeted food, as the introduction of acid/alkali may lead to adverse effects and is difficult to remove during later stages of the process (Zhang et al. 2022). At the same time, protease hydrolysis lacks the simulation of microbial action and can not truly interpret the human microenvironment. Although there is currently no research specifically focused on optimizing the enzymatic hydrolysis process for Lophius little peptides, we can draw parallels from studies on collagen peptides and muscle proteins of marine fish (Foh et al. 2010). For instance, the auxiliary enzymatic hydrolysis technology used for tuna and herring peptides, as detailed in Table 1, can help to avoid the introduction of interfering factors and improve the enzymatic hydrolysis efficiency of neutral protease.

### 2.2 Arthropod Gate

#### 2.2.1 Shrimp

Shrimp peptides are mostly derived from Antarctic krill, with a small portion obtained by enzymatic hydrolysis of protein hydrolysates from the shell and head of northern shrimp ( *Pandalus borealis* ) to obtain a peptide mixture “refined shrimp peptide concentrate” ( *Pandalus borealis* ) (Li, Cheng, et al. 2020; Li, Lv, et al. 2020). As shown in Figure 2C, Antarctic krill, and Chinese shrimp are currently focused on preparing shrimp products like shrimp peptide calcium, which is highly popular among consumers. Antarctic krill peptides have been found to possess health effects such as enhancing immunity, anti‐fatigue, and preventing osteoporosis. From a nutritional standpoint, Antarctic krill protein can provide all essential dietary amino acids. It was once considered to be a valuable resource for various foods and medicines, promoting the absorption and utilization of amino acids (Chen et al. 2009). For example, Antarctic krill protein can form a chelating complex with Ca, which can promote the absorption of calcium in the intestine and improve the bioavailability of calcium in the human body (Wang et al. 2015), thus nourishing the intestinal microbial environment and enhancing the intestinal mucosal immunity. In terms of functional regulation potential, many important physiological and biochemical functions in the organism are affected by activating the protein expression of the pathway and affecting the neuromodulators (Ognik et al. 2020). For instance, in liver protection, Antarctic krill peptides (AKP) exert a protective effect against acute liver injury (ALI) in mice by activating the protein expression on the Nrf2/HO‐1 single pathway. In the study of immune diseases, according to the experimental results, shrimp peptide hydrolysate (SPH) demonstrates immunomodulatory effects in immunosuppressive mice induced by cyclophosphamide (Khan et al. 2022). The evaluation of the experimental results regarding the intestinal microbiota shows that the shrimp peptide hydrolysate restores the ecological balance of the intestinal microbiota by removing various harmful microorganisms and promoting metabolism to enhance immunity. At the same time, it also increases immune organ index, serum cytokine levels (IFN‐γ, IL‐1β, TNF‐α, IL‐6), and immunoglobulin levels (IgA, IgM) (Khan et al. 2022). It is evident that shrimp peptide hydrolysate can be used as a functional food, serving as an immune function regulator to increase the practical significance of the immune organ index to promote intestinal health, and can be used as an auxiliary treatment in the later stage of cyclophosphamide drugs.

It is well known that the immunoactive substances in food are only trace amounts, but can be used as a dietary supplement to alleviate the damaged immune system. In the preparation process, the main concern is the enrichment of active ingredients in dietary supplements. As shown in Table 2, during the preparation of shrimp protein and the extraction of shrimp by‐products, the head of white shrimp can degrade tissue protein by autolysis (Cao et al. 2008), allowing for the recovery of protein from shrimp head waste at a low cost. However, many endogenous enzymes are inactive and act slowly in the process of autolysis (Cao et al. 2014). Therefore, to enhance shrimp protein stability and ensure consistent activity of shrimp peptides, ultraviolet irradiation can be employed to activate and maintain endogenous enzyme activity in shrimp processing by‐products, thereby improving protein extraction efficiency (Cao et al. 2014), which not only reduces the cost but also improves the preparation efficiency. However, excessive UV irradiation can easily lead to protein denaturation and enzyme inactivation (Fernandes et al. 2012). Ultrasonic technology remains very helpful for the preparation of shrimp products. For example, ultrasonic technology is used to extract flavor compounds and proteins from shrimp heads. While there are fewer studies focusing on protein extraction, the technique is primarily used for the extraction of flavor compounds. However, the enzymatic hydrolysis of rapeseed protein (Wang et al. 2016) and rice protein (Xie et al. 2024) has been studied with significant effects. Studies have shown that ultrasound can accelerate the degree of hydrolysis by altering the spatial structure of the substrate protein into a loose and stretched state, exposing the active sites buried inside the protein (Yan et al. 2025; Zhang et al. 2017), which is conducive to better structural modification and design of metal ion chelating peptides. Additionally, it can also destroy hydrogen bonds and hydrophobic interactions, altering the conformation of the protein to make it more accessible for enzymatic hydrolysis. Ultrasound‐assisted technology is not only beneficial to the affinity of the protein itself but also helps to enhance protease activity. Research shows that ultrasonication and high‐pressure homogenization resulted in comparably high protease activities in the BSY extracts produced (Schottroff et al. 2025). Therefore, ultrasonic‐assisted enzymatic hydrolysis can be utilized in the preparation of immunoreactive peptides to improve protein yield and protease activity.

### 2.3 Echinoderms

#### 2.3.1 Sea Cucumber

As shown in Figure 2D, sea cucumber proteins are primarily derived from the body wall (approximately 90%, polypeptides per dry body wall of sea cucumber) (Pangestuti and Arifin 2018; Cusimano et al. 2019). Currently, bioactive peptides are considered important potential therapeutic substrates in sea cucumbers. As shown in Table 1, enzymatic hydrolysates from the viscera of Atlantic sea cucumbers have been reported to possess antiviral and antioxidant activities (Pangestuti and Arifin 2018). For example, sea cucumber hydrolysates have been shown to have antiaging effects (Chen et al. 2025). The primary research focus is on how to increase the abundance of beneficial bacteria and reduce harmful bacteria, thereby regulating the intestinal microbial environment. Especially for the regulation of the body's immune system, dendritic cells are prompted to recognize intestinal bacteria through TLR, activate signaling pathways, and produce different inflammatory or immune cytokines. Thus, regulating the differentiation of T cells into different subsets, achieving the balance between pathogenic microbial tolerance and immunity, and alleviating functional disorders caused by immune dysfunction. In the study of immune diseases, some studies have found that sea cucumber peptides can up‐regulate the immune response of T lymphocyte subsets by regulating the diversity of intestinal microflora, increasing the proportion of CD4^+^/CD8^+^ T lymphocytes, and showing potential antiallergic activity in ovalbumin allergic mouse models (Wan et al. 2020). Japanese artemisia hydrolysate (EH‐JAP) and white taro hydrolysate (EH‐LEU) have demonstrated the capability to alleviate diet‐induced hyperuricemia and kidney inflammation in mice. These hydrolysates also exhibit the potential to decrease the abundance of opportunistic pathogens, such as *Porphyrobacteria* and *Bacteroidetes*, which help in alleviating the dysfunction of intestinal microflora in hyperuricemia mice (Wan et al. 2020). Since the inflammatory response is closely related to the immune system, sea cucumber peptides can inhibit the intestinal inflammatory response by reducing the release of inflammatory cytokines and inflammatory factors (Ismail et al. 2025), which supports the promotion of immune tolerance in a stable environment and avoids excessive immunity or immune tolerance. Sea cucumber peptides' anti‐inflammatory activity and antioxidant activity hold promise for their development into immunoreactive peptides.

Combined with Tables 1 and 2, it becomes apparent that most of the neutral protein hydrolysates of sea cucumber peptides have anti‐inflammatory and antioxidant activities, while the papain hydrolysates of the sea cucumber body wall also exhibit antiproliferative effects on HepG2 and potential for targeted drug delivery. Papain, being a thiol‐containing protease, can hydrolyze amide and ester bonds, cleaving peptide bonds composed of residues of arginine, lysine, and phenylalanine. Bromelain, on the other hand, cleaves at the carbonyl ends of glycine, tyrosine, alanine, and lysine. However, the combination of enzymes shows stronger antioxidant potential than single enzymes and untreated enzyme (Ding et al. 2025). The combination of two proteases is more conducive to the quality and efficiency of sea cucumber protein hydrolysates. The hydrolysates of bromelain and papain contain higher concentrations of essential amino acids and high antioxidant capacity (Choe et al. 2006). Glutamic acid, glycine, and alanine related to immunomodulatory activity were identified as the most important umami components in seafood (Zhang et al. 2022), making it possible to consider consumer experience while ensuring the biological activity of the peptides. In the production and preparation of immunoreactive peptides, it is crucial to distinguish the enzymatic hydrolysis performance and selectivity of each protease. Selecting the appropriate protease combination can effectively improve the quality and efficiency and may even improve the postoperative rehabilitation effect.

### 2.4 Mollusk Door

#### 2.4.1 Oyster

The immunomodulatory and anticancer effects of oyster immunoreactive peptides have been confirmed (Chalamaiah et al. 2018; Zhao et al. 2014). As shown in Table 1, oyster protein hydrolysates have been reported to enhance the killing ability of immune cells by enhancing spleen lymphocyte proliferation and macrophage phagocytosis, thereby enhancing the clearance of pathogens and playing an immune protective role in BALB/c mice (Wang et al. 2021). Oyster peptides (OP) also demonstrated the same immunomodulatory effect as shrimp peptides on intestinal immunosuppression in cyclophosphamide‐treated mice. According to the study results, OP significantly increased the index of immune organs, restored the integrity of intestinal mucosa, stimulated the secretion of cytokines (IL‐2, IFN‐γ, IL‐4, and IL‐10) and sIgA in serum, and alleviated the intestinal inflammatory injury induced by cyclophosphamide by activating the NF‐κB pathway (Xiang, Zheng, et al. 2021; Xiang, Zhou, et al. 2021). Therefore, OP can be used as a potential health‐promoting regulator of the gut microbiome or an immunomodulator in food or medicine. Interestingly, some researchers have found a complex relationship between intestinal microflora and fatigue‐related biochemical indicators. For example, oyster immunoreactive peptides can alleviate fatigue caused by body damage in the later stage of chemotherapeutic drugs (Xiao et al. 2020). Fatigue can be reduced and immune system damage can be improved by improving the environment of the intestinal microbiota.

Combined with Tables 1 and 2, oysters are of significant interest to consumers. The hydrolysate of oysters not only exhibits antitumor activity and immune stimulation in BALB/c mice (Wang et al. 2010) but also its oyster shell displays anti‐inflammatory properties. Oyster proteins with various activities depend on different protease hydrolysis; for example, oyster hydrolysates produced by pepsin treatment have higher anticoagulant activity (Gale et al. 1997). Although the enzymatic hydrolysis of oyster protein is superior to other hydrolysis methods, complementary techniques can be employed to further optimize the preparation process. Studies have shown that mussels, which are also shellfish marine organisms, benefit from pulsed electric field treatment, which significantly increases protein yield (Zhao et al. 2021). It is worth noting that pulsed electric field treatment changes the secondary and tertiary structures of proteins, leading to the loss of α‐helix and β‐helix (Bhat et al. 2019). For the preparation of oyster products, moderate hydrolysis is often sufficient; thus, high hydrostatic pressure treatment can be used, which is gentler than pulsed electric field treatment and can also extend the physical, microbial, and chemical quality of oysters (Tabatabai and Deyoe 1984). In the food field, glycosylation can induce multi‐level changes in protein structure by directly modifying hydroxyl groups (O‐glycosylation) or indirectly changing the hydroxyl microenvironment, thereby regulating functional properties (such as solubility, stability, texture) in food processing. For instance, glucose‐glycosylated oyster protein has the best gel properties (Li et al. 2023), and the preparation process of oyster gelatin candy can be further optimized to improve the yield per unit protein.

Through the optimization analysis of the preparation process of marine animals with immunomodulatory activity, molecular docking technology has research significance for analyzing protein binding sites in the field of food and medicine and food homology. It can be combined with the response surface method to improve the efficiency of experimental design. Interestingly, immune proteases with immune activity maintain cell homeostasis by degrading defective proteins and play a role in T cell expansion and differentiation. It can be seen that this immune proteasome helps to shape the ideal tumor microenvironment and plays a synergistic role in cancer treatment (Chen et al. 2023). This provides a novel approach for the design of the damaged intestinal model delivery system. In addition, in some studies, pulsed electric fields, ultrasound, and microwave‐assisted methods have been used to promote the efficiency of enzymatic hydrolysis. It is particularly noteworthy that some nonthermal methods may lead to protein denaturation and affect protein activity. Despite the mild reaction conditions for enzymatically catalyzed oligopeptide hydrogel formation, it exhibits good regional and stereoselectivity. However, in the process of protein food use, the slow‐release performance of the hydrogel cannot prevent the protein from being degraded and inactivated by the protease. Current studies have shown that, as shown in Figure 4B, nanocapsules can shield the degradation activity of proteases on loaded proteins, and small molecule precursors are more likely to penetrate the polymer network and enter the catalytic pocket of proteases to self‐assemble into hydrogels. It not only effectively prevents proteolysis but also facilitates the dissolution of effective substances and accurately regulates the intestinal microenvironment (Zhao et al. 2014).

## 3 Regulation Mechanism of Potential Immunoreactive Fragments

### 3.1 Intestinal Flora Regulates the Immune System

As shown in Table 3, gut microbiota regulates immune development through the interaction of metabolites (SCFAs, lactic acid, etc.) and immune cells. Among them, the metabolites of key flora (such as Bifidobacterium, Bacteroides, and Firmicutes) are the core media connecting the flora and the immune system, and their dysfunction may lead to increased susceptibility to autoimmune diseases or infections. It can be seen that the gut microbiota plays a crucial role in the development of the host's innate and adaptive immune systems. It can stimulate immune cells and promote the release of inflammatory factors and immune cytokines, effectively treating diseases and alleviating damage originating from the intestinal tract. Food‐derived marine immunoreactive peptides have a profound impact on the balance of tolerance and effector immune function by regulating the specific immune response of intestinal flora to antigens. Intestinal flora can affect the immune system by regulating the activity and function of immune cells. The composition and function of intestinal flora play a critical role in regulating the metabolism and function of immunoreactive peptides. The functional status of flora also affects the mechanism and efficacy of immunoreactive peptides. Different flora composition may lead to different metabolic pathways and metabolites, which will also affect the anti‐inflammatory effect of active peptides. For example, fecal bacteria can produce butyrate, which can improve the inflammatory environment of the intestine, thereby alleviating inflammation and promoting immune response. Marine immunoactive peptides are conducive to promoting the growth of beneficial bacteria in the intestine. Anglerfish peptides increase the abundance of *Bacteroides* and lactic acid bacteria and increase the activity of beneficial bacteria such as lactic acid bacteria and bifidobacteria, thereby augmenting the activity of immune cells (Cui et al. 2022) and stimulating the immune response. Studies have shown that immunoreactive peptides regulate the intestinal immune microbial environment and inhibit the release of inflammatory factors and opportunistic pathogens. Additionally, marine active peptides can also strengthen the intestinal epithelial cell barrier and reduce the entry of harmful substances, thereby diminishing inflammatory lesions and enhancing the immune system.

**TABLE 3:** Effects of intestinal flora and its metabolites on intestinal immune regulation.

Intestinal flora	Metabolite	Types of immunocytes	Functional effect	Influences on the immune system	References
Bifidobacterium	Lactic acid, acetic acid bacteria exopolysaccharides (EPS), peptidoglycan	CD4^+^T cells, DC	Enhance mucosal immunity, lgE↓	It can produce antibodies, and cytokines, and increase the intestinal mucosal barrier and the immune response	Liang et al. 2025
Acidobacterium	lactic acid, lactic aldehyde, lactic aldehyde, lactic ester, etc	DC, Inhibition of the CD8^+^T cells	Specific immunity, IgG↑	Improve the immune system by regulating immune cytokines and anti‐inflammatory	Feng et al. 2022
Bacteroidetes	Various organic acids, enzymes, amino acids and other compounds, short‐chain fatty acids (acetic acid, propionic acid)	T cells, DC and macrophages	Regulation of immune cell differentiation (such as Th17) enhances mucosal defense, slgA↑	Inhibiting maturation and regulated production of cytokines or chemokines, regulating CD4 + T cell differentiation and cytokine production, and memory CD8 + T cell response induction.	Parker et al. 2020
Firmicutes	A variety of organic acids, enzymes, antibiotics, enzyme inhibitors, short‐chain fatty acids (butyric acid)	T cells, DC and macrophages	Enhance mucosal immunity, IgA↑、lgE↓	Short‐chain fatty acids (butyric acid) can limit the synthesis and secretion of pro‐inflammatory cytokines, and maintain the renewal ability of immune cells in the intestinal mucosa	Ma et al. 2020
Fusiformis	Lactic acid, butyric acid, propionic acid, acetate, gas, and cholesterol, and short‐chain fatty acids	DC, T cell, Tregs	Regulate the activity of intestinal immune cells, IgA↑	It suppresses the growth of pathogenic bacteria and regulates the activity of T cells and macrophages in the intestine, thus affecting the balance and function of the immune system	Jafari et al. 2014

#### 3.1.1 Bifidobacterium and Its Metabolites Short‐Chain Fatty Acids

Bifidobacterium forms a macro‐control of the intestinal microenvironment through four mechanisms: immune regulation (Treg/IL‐10), barrier strengthening (mucus/tight junction), metabolic inhibition (SCFAs/lactic acid) (Liang et al. 2025), and competitive rejection. Its role depends on the dynamic interaction of immune active substances‐bacteria‐host‐metabolites, which provides an important target for the prevention and treatment of intestinal diseases. Studies have found that bifidobacteria can increase the expression of IL‐10 in intestinal Treg cells, thereby enhancing the T cell response and immune response. By enhancing the immunosuppressive effect of Treg cells, bifidobacteria can alleviate the intestinal immune side effects caused by CTLA‐4 blockade (Lo et al. 2024). At the same time, intestinal dendritic cells (DC) produced by bifidobacteria promote the production of IL‐6, TNF‐α, and IL‐12 (Bamias and Cominelli 2016). However, it is worth noting that the immunomodulatory properties are strain‐dependent (Fink and Frøkiaer 2008), especially as *Bifidobacterium* affects bacterial‐dependent cytokine production. Therefore, further evidence is needed to elucidate the specific immune responses elicited by each bifidobacterial strain in the intestinal mucosa. In the study of exploring the immune active substances to improve the immune regulation of intestinal flora, it is essential to determine the specific adaptive immune response of strains in the intestine. For example, the TLR2‐ERK/MAPK/NF‐κB signaling pathway is specifically involved in the maturation and polarization of innate immune cells (Chuang et al. 2007). Food‐derived marine immunoreactive peptides, when fed to the gut microbiota, can modulate the adaptive immune regulation of probiotics, transducing signals to the Treg/Th17 axis (Duan et al. 2023). Some studies have found that marine immunoreactive peptides can increase the number of bifidobacteria in the intestine, thereby promoting the production of SCFAs, maintaining the integrity of the intestinal mucosa, and enhancing the function of intestinal epithelial cells to regulate the intestinal immune response and promote nutrient absorption.

Short‐chain fatty acids (SCFAs), including acetate, propionate, and butyrate, act as key mediators between the intestinal microbiome and the immune system, playing a vital role in maintaining both the innate and adaptive immune systems (Wang et al. 2021). Oyster peptides, sea cucumber peptides, and tuna oligopeptides have the effect of increasing intestinal beneficial bacteria and SCFAs and can provide energy to intestinal epithelial cells, promote their growth and repair, and further enhance intestinal barrier function. SCFAs have important immunomodulatory functions, especially butyrate. Butyric acid can improve the structure of the flora, play an important role in the health and function of the intestinal barrier, improve intestinal immunity, and maintain intestinal homeostasis (Jiao et al. 2020). SCFAs can not only be absorbed in the intestine and maintain the integrity of the intestinal epithelial mucosal barrier but also be an effective anti‐inflammatory compound that can reduce the production of cytokines by inhibiting NF‐κB (Zheng et al. 2021). Moreover, the metabolites produced by intestinal microorganisms can be absorbed by the colonic mucosa and influence the body's physiological processes through endocrine or metabolic pathways involving bile acids (Sweeney and Morton 2014). Cellular and animal experiments with tuna eggs have demonstrated that tuna egg protein increases the production of gut‐derived metabolites (such as indolepropionic acid [IPA] and SCFAs), exhibits antioxidant activity, enhances the phagocytic and bactericidal abilities of macrophages, boosts the activity of natural killer cells, and improves antiviral immune responses. Butyrate supplementation (Jirsova et al. 2019) has been shown to increase the expression of tight junction proteins and lysozyme in the ileum mucosa, which partially alleviated the intestinal barrier damage induced by parenteral nutrition supplementation (Chapman et al. 1995). Additionally, SCFAs can also promote the migration of immune cells, enhance the ability of immune cells to remove infectious pathogens, and help regulate the host immune response (Zhan et al. 2022). It is evident that the immunoreactive peptide increases the production of SCFAs and their metabolites while supplementing enteral nutrition, which helps to improve the intestinal immune microenvironment and enhance the ability of the immune response.

#### 3.1.2 Mechanism of Lactobacillus Improving Immune Microenvironment

*Lactobacillus acidophilus* and *Lactococcus* are common lactic acid bacteria known for their antibacterial, anti‐inflammatory, and immunomodulatory properties (Amdekar et al. 2013). Most importantly, the *Lactobacillus acidophilus* lysate has been shown to inhibit M2 polarization and IL‐10 expression in LPS‐activated RAW264.7 macrophages (Wu et al. 2013), which provides a novel idea for studying the inhibition of intestinal flora on intestinal cancer diseases. *Lactobacillus* reduces pathogen colonization by blocking pathogen adhesion to epithelial cells, such as the use of siderophore‐based immune delivery strategies to present antibodies and reduce pathogen colonization (Iatsenko et al. 2020). Thereby, it inhibits pro‐inflammatory cytokines such as IL‐1 and IL‐6 and promotes the production of regulatory cytokine IL‐10, reducing the inflammatory response. For the intestinal barrier, acid‐resistant *Lactobacillus* can enhance the integrity of the intestinal mucosal barrier and reduce the penetration of harmful substances. It can increase the secretion of intestinal mucus, forming a protective barrier against damage from harmful substances and regulating the function of the intestinal immune system to enhance immunity. Additionally, it can promote the activity of immune cells, increase the production of immunoglobulin, and improve the body's resistance to pathogenic bacteria. In recent years, the potential molecular regulation mechanism of *Lactobacillus acidophilus* has been gradually studied. *Lactobacillus acidophilus* can prevent or treat IBD by regulating immune response, restoring intestinal microflora and other ways (Kim et al. 2021), as well as alleviating intestinal injury caused by chemotherapy drugs. *Lactobacillus* and its products play an important role in intestinal flora and host health. It is not only the decomposition process of dietary supplements in vivo but also the use of *Lactobacillus acidophilus* and other flora for in vitro fermentation. For example, the fermentation of a mixture of *Ganoderma lucidum* and probiotics can regulate the immunosuppression of cyclophosphamide (Li et al. 2023), which offers a new idea for the fermentation of traditional Chinese medicine (TCM) as a dietary supplement by probiotics. Sea cucumber peptides and angler fish protein hydrolysates can increase the abundance of beneficial lactobacilli and SCFAs and reduce the abundance of opportunistic pathogens to alleviate the dysfunction of the intestinal microflora, improve the immune system by promoting the function of immune cells, and maintain intestinal health. For example, Lophius little peptides enhanced intestinal microbial diversity by improving the proportion of *Firmicutes* and *Bacteroides*, especially increasing the abundance of *Bacteroides* and *Lactobacillus* (Xu et al. 2025).

Cloning and functional studies of proline‐rich AMP (PR‐AMP) from three mud crabs revealed that peptidoglycan stimulated SpPR‐AMP1 in blood cells and up‐regulated mRNA expression levels. Furthermore, SpPR‐AMP1 promotes the growth of beneficial bacteria such as *Lactobacillus* and exerts immunomodulatory effects. The human innate immune system uses bacterial peptidoglycan (PG) recognition elements to respond to pathogens and commensal bacteria at the molecular level (Imjongjirak et al. 2017). It can be seen that proline‐enriched AMP immunoactive peptides can destroy the membrane structure of pathogens and are induced by PAMP (such as peptidoglycan), reflecting the specificity of the immune response and synergistically enhancing host defense. Peptidoglycan fragments, such as cell wall acyl dipeptides, released into the extracellular environment participate in various biochemical activities, including cross‐species signal transduction and stimulation of the immune system. These fragments can enter the host cytoplasm, where they are recognized and bound by specific receptors, Nod1 and Nod2, triggering the host's immune response. Previous studies have suggested that the antitumor effects of Lactobacillus may be attributed to the activation of macrophages by PG expressed on the surface of the bacterial cell (Saito et al. 2024). The enteral administration of peptidoglycan can be designed to target these effects, which could have implications for monitoring the efficacy of dietary supplements on intestinal flora in the future (Saito et al. 2024).

### 3.2 Regulating the Immune System by Amino Acid Metabolites Decomposed by Intestinal Flora

Marine active peptides play a significant role in enhancing the production of signal molecules by influencing intestinal flora. They can regulate immune cell activity and immune response levels, as well as decrease both the intensity and duration of inflammatory responses. Correspondingly, protein serves as the sole carbon source for the synthesis of SCFAs or branched‐chain fatty acids (BCFAs) outside the intestinal flora (Sam et al. 2021), providing nutrients for the intestinal flora. Different intestinal flora species can produce distinct SCFAs through the fermentation of different amino acids. For instance, acetic acid can be produced by the fermentation of glycine, alanine, threonine, glutamic acid, lysine, and aspartic acid (Li et al. 2025). In addition, it can also produce butyric acid by fermentation of alanine, which has a certain regulatory effect on the tumor microenvironment. Butyrate, produced by SCFAs, can enhance the efficacy of anti‐PD‐1 monoclonal antibodies by infiltrating T cells. Research into marine immune‐active peptides has revealed that the crust protein of *Penaeus vannamei* , as an immune effector, is rich in cationic cysteine antimicrobial peptides, which are beneficial in resisting pathogen invasion (Li et al. 2025). At the same time, proline‐rich residues or glycine‐rich active peptides are helpful for further study of immune active sites. Because its proline‐rich residues stabilize the functional structure of immune active sites through conformational constraints, and the flexible conformation of glycine endows dynamic adaptation ability, in macrophage‐related intestinal inflammation and tumor research (Wang et al. 2015), the analysis of its structure–function relationship can provide a key theoretical basis for drug design targeting immune active sites (such as small molecule inhibitors or antibody engineering), and open up new ways to intervene in pathological immune responses (such as excessive inflammation or immune tolerance). Peptides extracted from pepsin‐hydrolyzed sturgeon muscle exert anti‐inflammatory effects on LPS‐stimulated RAW264.7 macrophages through MAPK and NF‐κB pathway (Rauen and Tidyman 2024). After intestinal degradation, the C‐terminal of sturgeon peptide contains arginine and lysine, and the N‐terminal hydrophobic amino acids such as alanine, valine, and leucine, which may promote the binding of the peptide to the membrane and regulate the downstream inflammatory signaling pathway (Gao et al. 2020). Interestingly, the perch anti‐inflammatory active peptide has antibacterial activity against Gram‐negative bacteria, which may contribute to the homeostasis of the intestinal flora and have a positive impact on the pathogenesis of inflammatory diseases. It can clearly be seen that the anti‐inflammatory peptides, antimicrobial peptides, and antioxidant peptides of animal‐derived marine active peptides have certain effects on immune cells such as macrophages. Amino acids and their metabolites produced by intestinal microflora promote immune response and secretion of immune cytokines by regulating immune cells such as macrophages. At the same time, the antibacterial activity of perch peptide can be used to improve the intestinal immunity of patients during chemotherapy. It can not only accurately treat the tumor immune microenvironment, enhancing T cells and inhibiting tumor cells from outside the intestine but also regulate the intestinal flora microenvironment from the intestine to improve the damaged immune system and prevent the occurrence of postoperative complications such as inflammation. Marine active peptides can modulate both inflammatory and immune responses, which is crucial for maintaining the balance and stability of the immune system. This regulation is particularly important for preventing or assisting in the treatment of immune‐related diseases that may occur during cancer therapy. Due to the low immune regulation ability of the damaged intestinal microenvironment caused by disease and postoperative, it is urgent to balance the intestinal flora with marine active peptides composed of immune‐active amino acids to maintain the intestinal immune system. Studies have shown that threonine in food‐derived marine immunoreactive peptides can rebalance the intestinal microbiota. Supplementation of low crude protein with threonine can restore intestinal microbial diversity and significantly increase the abundance of potentially beneficial microorganisms in the body (such as *Bacteroides*, and *Enterobacter*) (Christovich and Luo 2022; Fusco et al. 2025).

### 3.3 Regulating the Immune System by Repairing the Intestinal Mucosal Barrier

**FIGURE 3:**
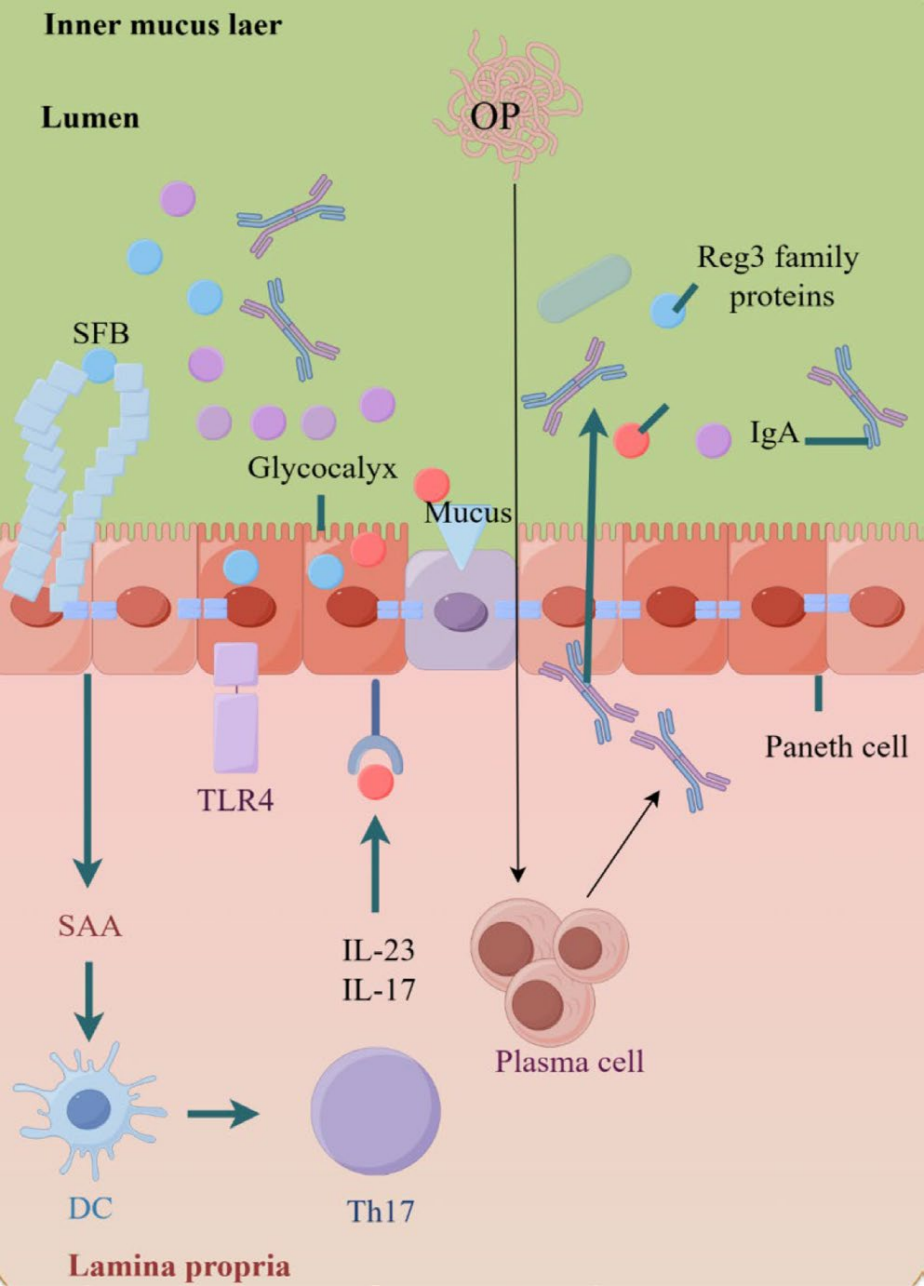
Immunoactive peptides stimulate immune cells to maintain the intestinal mucosal barrier (by Figdraw).

The integrity and permeability of the intestinal barrier are key defense lines to maintaining the body's homeostasis. An imbalance not only leads to local intestinal lesions but also affects systemic health through immune, metabolic, and neuroendocrine networks. Marine active peptides can promote the repair and reconstruction of intestinal barrier function. Immunoactive peptides can enhance the structure and function of intestinal epithelial cells, increase the integrity of the mucosal barrier, and reduce the penetration of harmful substances. Impaired intestinal epithelial barrier function in intestinal inflammation will lead to increased intestinal epithelial permeability and participate in the pathogenesis of IBD. There are several mechanisms by which marine active peptides can repair the intestinal mucosal barrier. The enhanced glycolysis (Warburg effect) caused by the antibacterial mechanism provides energy for immune cells (Chelakkot et al. 2023), and the anti‐inflammatory mechanism promotes fatty acid oxidation through the AMPK/mTOR pathway to maintain the homeostasis of immune cell function (Tang et al. 2023). Another mechanism involves adjusting the protein structure and function to improve the integrity of intestinal mucosal permeability. For example, low methionine can improve the function of the intestinal mucosal barrier by changing the structure and function of tight junction proteins and promoting the repair of damaged intestinal mucosa (Ognik et al. 2020). Tuna peptide has an intestinal protective effect on ulcerative colitis. It can improve intestinal flora disorder and enhance the protective effect of the intestinal mucosal barrier by promoting the secretion of SCFAs. In recent years, studies have found that food‐derived anti‐inflammatory active peptides mainly improve intestinal epithelial barrier function by repairing epithelial cells and tight junctions between cells (Fu et al. 2022), and avoid intestinal mucosal barrier damage by repairing intestinal barrier function. For example, Lophius little peptides strengthen the intestinal epithelial barrier to reduce kidney inflammation (Chi et al. 2021). As illustrated in Figure 3, when the intestinal barrier function is normal, the tight junction regulates the cell bypass by regulating the intercellular space in adjacent cells, allowing nutrients to enter the intestinal epithelium freely, preventing the passage of intestinal bacteria, toxins, and inflammatory mediators, maintaining the integrity of the intestinal mucosal barrier protection function (Xiang, Zheng, et al. 2021; Xiang, Zhou, et al. 2021). In Figure 3, sIgA is the immunoglobulin of the small intestinal mucosa, which plays a key role in maintaining intestinal homeostasis and the balance of the immune system. Alaska Pollock peptide (APP) and Oyster peptide (OP) also influence the final differentiation of IgA B cells (Li, Gong, et al. 2019; Li, Wang, et al. 2019), thereby promoting the secretion of plasma cells and increasing the secretion of SIgA and IgA on the intestinal mucosa, and repairing the intestinal permeability damage caused by LPS. Among them, APP improves inflammatory damage by promoting the secretion of IL‐6 and IL‐10. As depicted in Figures 1 and 3, bioactive peptides can improve the intestinal flora of UC mice, regulate the balance of Th17/Treg, and inhibit the activation of TLR4 signaling pathway genes. Th17 stem cells can resist the decrease of activity caused by amplification and have the ability to assist the antitumor immune activity of CD8^+^ T cells. Therefore, T helper cells can be regulated by dietary immunoreactive peptides to improve the intestinal immune microenvironment. Through the “sterilization‐barrier‐immunity‐metabolism” multi‐dimensional regulatory network, the symbiotic bacteria function is maintained while inhibiting pathogen colonization, and a dynamic defense system is constructed in coordination with the host immune system to provide potential treatment strategies for intestinal infections and dysbacteriosis‐related diseases, such as mud crabs rich in proline‐rich AMP, have the function of maintaining the immune mechanism of intestinal flora and avoiding infection. Their extended spiral structure contains multiple Pro‐Arg‐Pro characteristic sequences. Some antimicrobial peptides also have O‐glycosylation sites on amino acid residues at specific positions, which are closely related to biological functions, and regulate the intestinal immune microenvironment by repairing the intestinal mucosal barrier (Nguyen and Tureček 2017).

## 4 Application Prospects of Foodborne Delivery Systems

The self‐assembled food delivery system breaks through the oral limitation of traditional peptides through molecular engineering and functional design, and achieves targeted delivery and synergy of immunoactive peptides. As in the cyclophosphamide‐induced intestinal mucosal injury model, self‐assembled sea cucumber peptide nanoparticles (particle size 120 nm, encapsulation efficiency > 90%) reduced serum LPS levels and repaired the intestinal barrier by increasing the secretion of MUC2 in goblet cells and up‐regulating the expression of claudin‐3. At the same time, they promote the proliferation of bifidobacteria, increase the concentration of SCFAs, and inhibit NF‐κB pathway‐mediated renal inflammation (Amdekar et al. 2013). It shows multidimensional intervention potential in intestinal flora regulation, organ damage repair, and tumor immunotherapy, and provides innovative strategies for functional food and precise nutrition intervention. By reviewing the preparation process and structure–activity relationship of food‐borne marine immunoactive peptides, the self‐assembled food delivery system of immunoactive peptides was further explored. Sustained release improves the retention time in the body and promotes full absorption of the gastrointestinal tract. By targeting the release and improving the biological activity function, the peptide is avoided from being enzymatically hydrolyzed in the gastrointestinal tract, and the stability of food‐derived immunoreactive peptide delivery is improved. Peptide‐drug conjugates in the context of cancer treatment, biologically active functional payloads are connected to tumor‐targeting peptides through connectors (Dharap et al. 2005), demonstrating significant clinical efficacy (Mendonça et al. 2024). However, these act on extraintestinal tumor cells and do not improve the damaged gastrointestinal immune system. Immune injury diseases still occur during and after drug treatment. In this regard, auxiliary measures should also be taken to improve organ damage and inflammation. Food‐borne immune active peptides, such as oyster peptides and shrimp peptides, have a protective effect on the immune system after taking cyclophosphamide. The whole food derivative of tuna and sea cucumber peptides can be used as functional products to alleviate kidney inflammation. Sea cucumber peptides and anglerfish peptides are species that regulate intestinal flora richness and reduce kidney inflammation (Cui et al. 2022). Therefore, marine bioactive peptides can enhance the intestinal mucosal immune system and alleviate organ damage caused by treatment by regulating the structure and composition of intestinal flora, increasing beneficial flora and immune factors. It is worth noting that the secondary damage of protein food to the immune‐injured intestine is due to multiple mechanisms, such as macromolecular escape, pro‐inflammatory metabolism, oxidative stress, and toxicity of specific components. By optimizing protein sources, processing methods, and delivery strategies, the intestinal burden can be effectively reduced, and the repair can be promoted. The composite hydrogel system was encapsulated with immunoreactive peptides to target the treatment of immune‐injured diseases. As a precise and effective dietary supplement, it acts on the immune microenvironment, stimulating T cells to improve the ability of autoimmune response, and inhibiting the proliferation of tumor cells after cancer surgery (Li et al. 2024). In the current study, whole‐food synergies found in the context of dietary patterns may lead to more effective nutritional research (Saito et al. 2024). Based on the research progress of marine immunoreactive peptides alleviating immune‐injured diseases through intestinal flora, the feasibility of a marine immunoreactive peptide food delivery system is prospected, with intestinal flora or amino acid metabolism as the target.

### 4.1 Delivery Systems of Metal Ion‐Chelating Peptides in the Food Field

**FIGURE 4:**
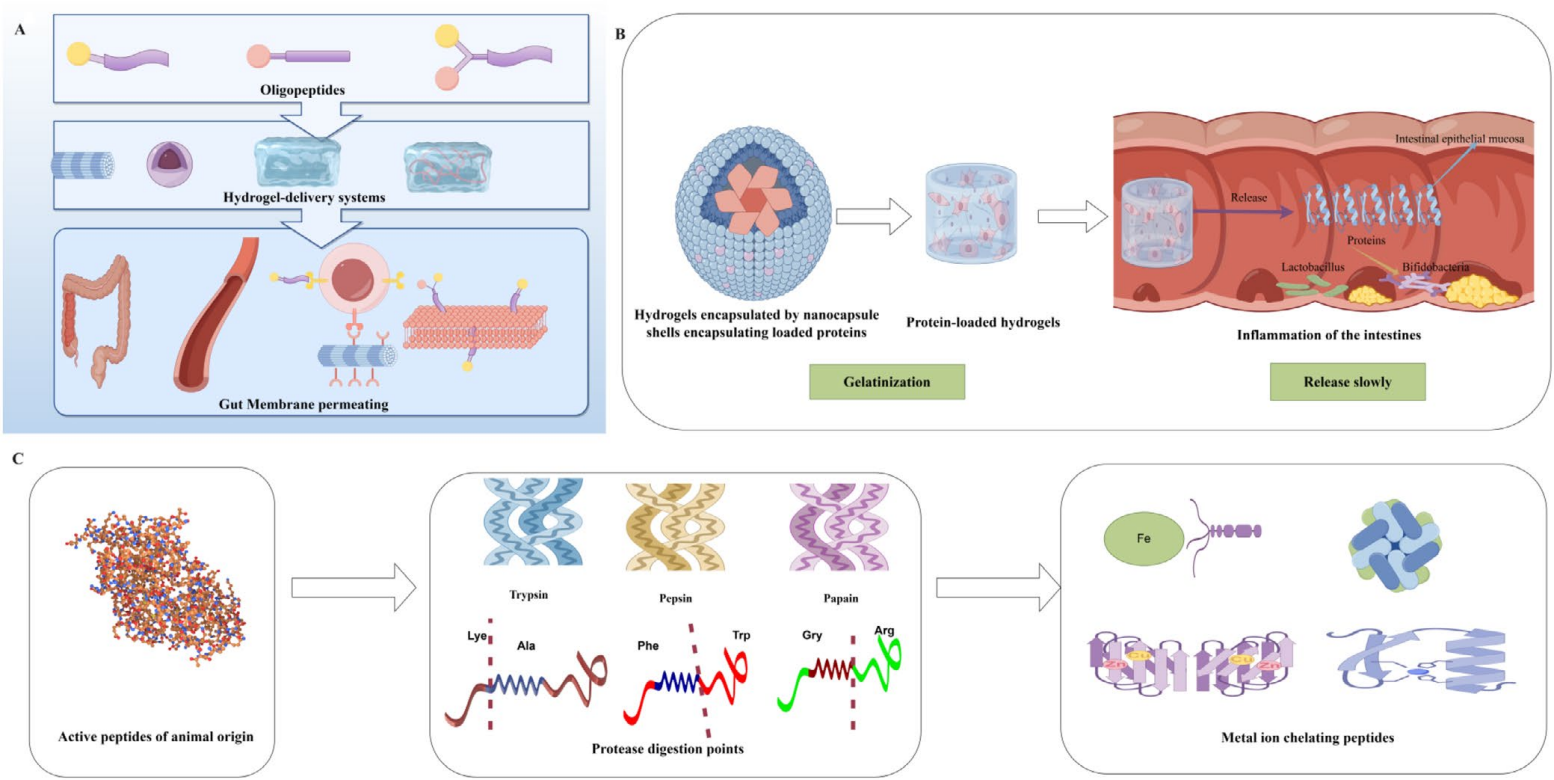
Intestinal delivery of immunoactive peptides and design of metal ion chelating peptide cleavage ligation sites (A: Loaded oligopeptide hydrogel delivery system design; B: Loaded nanocapsules encapsulated protein hydrogel intestinal release schematic diagram; C: Enzymatic hydrolysis and preparation of metal ion chelating peptides) (by Figdraw).

Amino acids have low toxicity, low immunogenicity, and high affinity for specific receptors in vivo. Their structural diversity, chirality, and multiple functional groups make them ideal ligands for the fabrication of nanomaterials for biomedical applications (Gogoi et al. 2019). Marine immunoactive peptides are rich in trace mineral elements, making them suitable for chelating and delivering with metal ions. The use of peptide‐metal ion chelates helps to avoid the direct damage of metal elements to the gastrointestinal tract and provides physiological and biochemical characteristics superior to inorganic metal ions. As shown in Figure 4C, the preparation of metal ion chelating peptides is to expose the active binding sites through natural proteases, which is more conducive to chelating with metal ions. However, dietary active peptides are susceptible to gastrointestinal proteases and have poor stability. Therefore, metal ion chelating peptides are combined with nanocapsules to avoid enzymatic hydrolysis and targeted delivery. Amino acid‐metal coordination nanomaterials (AMCNs) have shown great potential for biomedical applications in cancer treatment, antibacterial applications, etc., especially as drug carriers with specific tumor‐targeting capabilities. However, most nanocarriers lack hierarchical targeting ability, and their therapeutic indicators can be limited by poor tumor accumulation, low cell internalization efficiency, or inaccurate subcellular localization. The complexity of their design can hinder industrial development (Koczok et al. 2019). However, as a model of enteral administration, the specific recognition of marine animal‐derived immunoreactive peptides and peptidoglycan‐binding proteins of the intestinal flora cell wall alleviates the damaged intestinal immune microenvironment from the perspective of the intestinal flora, regulates the intestinal flora to enhance the intestinal mucosal immune system, and prevents complications and recurrence during tumor treatment. Based on the intestinal injury during tumor cancer immunotherapy, the enhancement of amino acid activity by amino acid‐chelated trace elements was explored. Immunoactive peptide multi‐amino acid synergy, multi‐target binding (Li, Gong, et al. 2019; Li, Wang, et al. 2019), aims to improve the stability of targeted drug delivery and enhance efficacy. It is safer and more effective than the self‐assembly system, which directly uses amino acids and metal ions.

The contents of glutamic acid, aspartic acid, and histidine have good metal ion binding ability, which indicates that Antarctic krill peptide, tuna peptide, sea cucumber peptide, oyster peptide, and anglerfish peptide are excellent protein peptide bases for preparing metal chelating peptides. In particular, the chelation of zinc ions with oyster protein hydrolysate (OPH) causes intramolecular and intermolecular folding and aggregation (Li, Gong, et al. 2019; Li, Wang, et al. 2019). Peptides rich in acidic amino acids (Glu and Asp) and basic amino acids (Lys, Arg, and His) are used as binding sites. The amino nitrogen atom of the carboxylic acid group and the oxygen atom of the carboxylic acid group are the primary binding sites of Zn (Geng et al. 2019). Glycine can also be chemically anchored on the surface of zinc through amino groups and exhibits a stable affinity for zinc, which enhances stability (Yang et al. 2023). The hydrophobic amino acid on the carboxyl donor side appears to increase its binding rate (Zhu et al. 2021). Studies have shown that Zn‐based immunoregulatory adjuvant (Zn‐LDH) can alleviate immunosuppression and achieve antitumor immunity without the assistance of cytokines or immune agonists (Wessels et al. 2021). Regarding the improvement of the intestinal microenvironment, the absence of zinc ions will lead to changes in intestinal flora diversity and SCFAs, change the abundance of intestinal mucin degradation‐related bacteria, and subsequently cause intestinal flora disorders, affecting the intestinal mucosal immune system. Conversely, excessive zinc can also result in a decrease in SCFAs. Therefore, combined with Table 2, when designing an immunoreactive peptide metal ion chelate delivery system, we should pay close attention to the effect of zinc ion content on intestinal flora and their metabolites such as SCFAs (Sun et al. 2024).

### 4.2 Design Based on Delivery System Predicts the Controlled Release of Hydrogel

Due to the specific cleavage of peptide bonds by proteases such as chymotrypsin, macromolecular marine immune peptides are decomposed into short peptides or amino acids (Rivett 1989), and the gastric acid environment may induce conformational changes of peptide chains (such as α‐helix unwinding), destroy key functional domains (such as receptor binding sites) (Morzel et al. 2023), resulting in loss of immune activity. At present, research on nano‐delivery active peptide systems has been carried out. The bioactive peptide is coupled to the nano‐delivery carrier through chemical bonds such as ester bonds and hydrazone bonds, which can not only prevent the peptide from enzymatic hydrolysis in advance but also improve the bioavailability of the drug (Xu et al. 2023). However, it is worth noting that the tissue concentration and blood concentration of the self‐assembly delivery system may be inconsistent due to the different targeting release location and release rate. Therefore, it is necessary to balance the targeting efficiency and blood clearance rate, and optimize the responsive release logic (such as dual pH/enzyme‐sensitive design). To ensure adequate absorption of the drug in the body, it is crucial to adjust the residence time and diffusion rate in the intestine, promoting the absorption of the active ingredient and minimizing loss. As shown in Figure 4A,B, the protein was encapsulated in the nanocapsules, and the hydrogel delivery system was used to release the controlled release through the permeation and diffusion of water to make up for this deficiency.

In medicine, stimuli‐responsive injectable hydrogels are employed to deliver the optimal amount of drugs to the target area within the required timeframe (Fu et al. 2023). Intelligent hydrogels with multi‐stimuli responsiveness and special functions have been widely used in drug delivery, particularly for polyamino acid hydrogels in tumor‐assisted immune regulation. The ultrasound‐responsive hydrogel delivers the internally loaded protein when triggered, and the protein release rate can be heightened by increasing the focused ultrasound amplitude and duration, demonstrating control over the stimulus–response mechanism (Singh et al. 2024). Nanomaterials can protect against excessive enzymatic hydrolysis of active peptides (Huang et al. 2025). The soft materials with unique three‐dimensional network structures formed by physical or chemical crosslinking of hydrogels can effectively maintain the stability of lipophilic encapsulated substances and achieve sustained release and controlled release in specific parts of the human body (Li, Du, et al. 2022; Li, Li, et al. 2022). Studies have shown that the aldehyde group of the hydrogel can covalently bind to amino groups on the intestinal mucosa, thereby stabilizing the adhesion of the hydrogel to the inner wall of the gut. This firm adhesion resists hydrodynamic shear forces, enabling sustained, low‐concentration drug release (Wu 1998; Zhang et al. 2024). The use of adhesion can maintain the effective delivery time of the hydrogel for sustained drug release, and can also significantly reduce the infiltration of intestinal inflammatory cells.

## 5 Summary

In conclusion, optimizing the enzyme preparation process or nonthermal assisted enzymatic hydrolysis technology to solve the limitations of natural proteolytic enzymes, that is, low protein yield. Delivery of food‐derived active peptides improves the intestinal immune microenvironment and increases the absorption rate of small peptides. This optimization includes the exposure of active amino acid binding sites to external media through a certain degree of physical catalysis and structural curl, which is more conducive to the design of enteral delivery systems. Similar marine active peptides in cell and animal studies have shown better efficacy by optimizing the enzymatic hydrolysis process. While optimizing the activity, it is also necessary to promote the functional transformation and product development of immunoactive peptides. Therefore, more evidence is needed to prove the protective effect of food‐derived immunoactive peptides as dietary supplements on intestinal and immune microenvironment immune injury, and to promote the innovative development of dual‐use products. It is worth noting that in the process of treating diseases, we should not only cure the disease but also minimize the damage of drug toxicity to the immune system, such as the intestinal mucosal barrier, and avoid ignoring the occurrence of various complications (Hamdeh et al. 2021). Supplementation of foodborne marine immune response peptides can improve the imbalance of intestinal flora and enhance the ability of the intestinal immune system to resist pathogen invasion. Previous studies have shown that oyster peptides can effectively improve cyclophosphamide‐induced intestinal immune injury in mice (Xiang et al. 2023). For the fragile intestinal immune system, the absorption rate of immunoactive peptides is crucial. Chelating delivery of active peptides with metal ions can improve stability and immune activity, improve postoperative intestinal microflora disorders and immune damage, and can be considered a postoperative functional adjuvant. Although more in‐depth studies are needed to confirm the value of active peptides in improving immunodeficiency, the delivery mechanism of food‐derived active peptides is a mild delivery method for intestinal dysfunction. Intestinal administration can regulate the intestinal microenvironment without damaging the damaged intestinal immune system, so that the immunoactive peptides can be fully absorbed into the body, making the food‐borne active substances more targeted, so as to improve the clinical postoperative rehabilitation effect and quality. This paper reviews the assisted enzymatic hydrolysis process of marine immunoactive peptides to increase the yield and improve the intestinal immune mechanism. The delivery mechanism in the food field has certain reference significance for the rehabilitation treatment of patients after clinical surgery. The targeting and effective absorption rate bring patients a good medication experience, which has certain guiding significance for the development of immunoactive peptide functional foods in the future.

## Author Contributions

**Zhicheng Yin:** conceptualization (equal), formal analysis (equal), investigation (equal), visualization (equal), writing – original draft (equal). **Yingying Tian:** conceptualization (equal), formal analysis (equal), resources (equal), visualization (equal), writing – review and editing (equal). **Shuteng Huang:** conceptualization (equal), formal analysis (equal), investigation (equal), visualization (equal). **Hong Wang:** formal analysis (equal), writing – review and editing (equal). **Lili Zhao:** formal analysis (equal), writing – review and editing (equal). **Ruyue Zhang:** formal analysis (equal), writing – review and editing (equal). **Desheng Cai:** conceptualization (equal), writing – reviewing and editing (equal), formal analysis (equal), visualization (equal). **Shuping Wang:** formal analysis (equal), writing – review and editing (equal). **Shaojing Zhong:** formal analysis (equal), writing – review and editing (equal). **Jiayu Zhang:** conceptualization (equal), formal analysis (equal), funding acquisition (equal), project administration (equal), supervision (equal), visualization (equal), writing – review and editing (equal).

## Conflicts of Interest

The authors declare no conflicts of interest.

## Data Availability

Data will be made available on request.
